# Annual Meetings of Four Children's Hospitals

**Published:** 1918-06-01

**Authors:** 


### Annual Meetings of Four Children's Hospitals.

### HOSPITAL FOR SICK CHILDREN.

Presiding at the recent sixty-sixth annual general court of governors of the Hospital for Sick Children, Great Ormond Street, Lord Aberdare stated that last year 2,792 in-patients and 25,297 new out-patients received attention. Despite a small decrease in annual subscriptions there was an increase of £3,998 in total income as compared with 1916. Expenditure, however, had gone up during 1917, exceeding that of the previous year by £1,911, or 7.6 per cent. A sum of £20,000 had been received from the estate of the late Mr. Peter Reid for the founding of a convalescent home in connection with the hospital. Other large donations included £1,000 from the estate of the late Mr. Leopold Salomons.

### ALEXANDRA HOSPITAL.

Provided a suitable site could be found it was decided at the annual general meeting of governors of the Alexandra Hospital for Children with Hip Disease, Queen Square, Bloomsbury, W.C., held under the chairmanship of Mr. W. H. Whitfield, to remove the institution to the country, and the committee of management was empowered to take the necessary steps in the matter. The hospital possesses ninety beds—sixty-eight in London and twenty-two at the country branch at Clandon, Surrey—and last year 142 children were under treatment, forty-six of whom had been discharged cured. Subscriptions last year totalled £943, as against £992 a year previously—a decrease of £49, but the income for 1917 exceeded the expenditure by £2,097.

### QUEEN'S HOSPITAL.

Colonel Lord William Cecil occupied the chair at the fiftieth annual meeting of governors of the Queen's Hospital for Children, Hackney Road. Owing to the difficulty in maintaining the necessary staff the number of in-patients decreased last year to 1,852, as compared with 2,161 in 1916. Nearly 7,000 more out-patients, however, received attention in 1917 than during the previous year. Their attendances had amounted to 111,806. The expenditure for last year had exceeded the income by £2,129. That amount was reduced to £1,585 by a credit balance brought forward from 1916. At the seaside branch—the "Little Folks'" Home, which contains thirty beds—258 children were admitted during the period under review, forty-five days being the average stay of the patients. The weekly cost of maintenance per patient had increased from 19s. 11d. in 1916 to £1 4s. 3d. last year.

### VICTORIA HOSPITAL.

Mr. Alfred Farquhar, the honorary treasurer, presided at the annual meeting of governors of the Victoria Hospital for Children, Tite Street, Chelsea. The number of patients treated during 1917 was 1,189, as compared with 1,294 a year previously, the decrease being due to the closing of some of the wards from time to time owing to the shortage of nurses. New out-patients last year numbered 13,679, with 64,809 attendances, as against 16,016 and 61,223 attendances in 1916. The income for 1917 amounted to £10,601 and the expenditure to £10,352, leaving a balance of £249 to be carried forward.

